# mRNA vaccines in the prevention and treatment of diseases

**DOI:** 10.1002/mco2.167

**Published:** 2022-08-25

**Authors:** Yangzhuo Gu, Jiangyao Duan, Na Yang, Yuxin Yang, Xing Zhao

**Affiliations:** ^1^ State Key Laboratory of Biotherapy and Cancer Center West China Hospital Sichuan University; Collaborative Innovation Center for Biotherapy Chengdu China; ^2^ Department of Life Sciences Imperial College London London UK; ^3^ Stem Cell and Tissue Engineering Research Center/School of Basic Medical Sciences Guizhou Medical University Guiyang China

**Keywords:** cancer, infectious diseases, lipid nanoparticles, mRNA delivery, mRNA vaccine

## Abstract

Messenger ribonucleic acid (mRNA) vaccines made their successful public debut in the effort against the COVID‐19 outbreak starting in late 2019, although the history of mRNA vaccines can be traced back decades. This review provides an overview to discuss the historical course and present situation of mRNA vaccine development in addition to some basic concepts that underly mRNA vaccines. We discuss the general preparation and manufacturing of mRNA vaccines and also discuss the scientific advances in the in vivo delivery system and evaluate popular approaches (i.e., lipid nanoparticle and protamine) in detail. Next, we highlight the clinical value of mRNA vaccines as potent candidates for therapeutic treatment and discuss clinical progress in the treatment of cancer and coronavirus disease 2019. Data suggest that mRNA vaccines, with several prominent advantages, have achieved encouraging results and increasing attention due to tremendous potential in disease management. Finally, we suggest some potential directions worthy of further investigation and optimization. In addition to basic research, studies that help to facilitate storage and transportation will be indispensable for practical applications.

## Graphical abstract

**Mechanisms of mRNA vaccines for infectious diseases and cancers**. mRNA molecules encoding tumor antigens are injected into body (either with or without delivery vehicles). The mRNA molecules are taken up and translated into protein antigens by antigen‐presenting cells (APCs). After proteasomal processing of proteins, antigen peptides associate with MHC Class I molecule in the endoplasmic reticulum and are transferred to the APC surface, activating CD8^+^ T cells for a specific cellular immune response. Protein antigens, which are sorted for the endosome route, can activate CD4^+^ T cells via the MHC Class II presentation pathway. The secretory protein antigen or membrane antigen encoded by mRNA can stimulate B cells to produce neutralizing antibodies and activate phagocytes such as macrophages to secrete inflammatory cytokines, facilitating the clearance of circulating infectious pathogens and tumor cells.

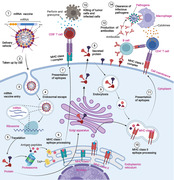

## 1 INTRODUCTION

Messenger ribonucleic acid (mRNA) vaccines are now regarded as an attractive and promising alternative to conventional vaccines. mRNA vaccines show several prominent advantages compared to live attenuated and subunit‐based vaccines.^1^, ^2^ First, they exhibit reliable potency in the induction of immune responses.^3^ mRNA vaccines are able to drive both humoral and cellular immunity.^4^, ^5^ In addition, RNA interacts directly with pattern recognition receptors (PRRs), driving a robust innate immune response without the need to involve additional adjuvants. However, innate antiviral responses may be a double‐edged sword, as they may reduce vaccine effectiveness by downgrading mRNA expression. Second, mRNA vaccines are safe, avoiding many risks of conventional vaccines, such as reversion to virulence or insertional mutagenesis.^6^, ^7^ Furthermore, mRNA, as a minimal genetic unit, does not stimulate any antivector immunity, making recurrent administration of mRNA vaccine safe and easy.^8^, ^9^ Third, mRNA vaccines are rapid and inexpensive to manufacture, because the synthesis of mRNA vaccines mainly uses in vitro transcription (IVT),^10^ which bypasses the time‐consuming steps of in vivo protein expression (i.e., cloning and cell culture).^11^, ^12^ Lastly, mRNA is a manageable transient modulator of physiologic processes.

**FIGURE 1:**
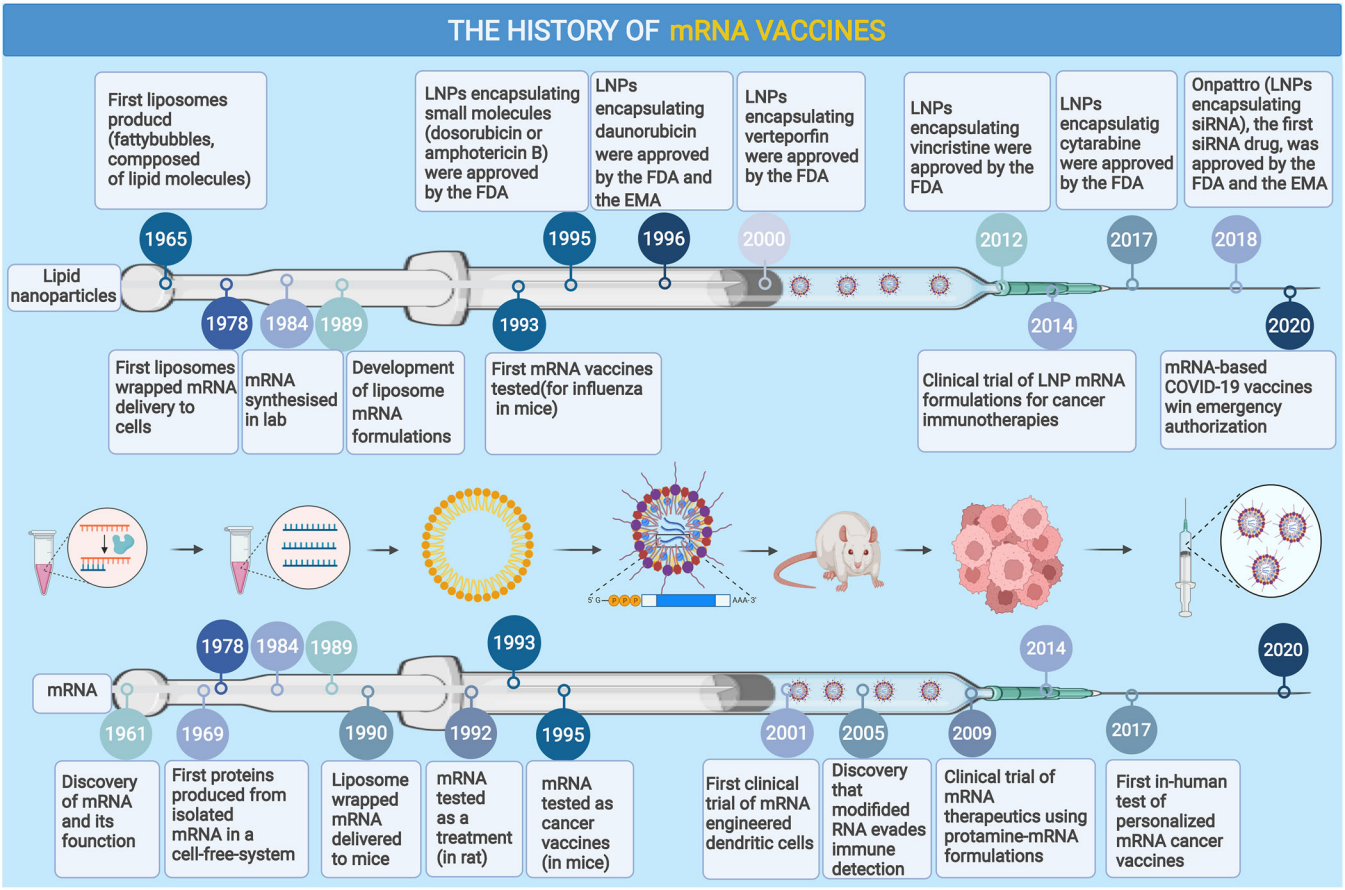
Timeline of major findings and breakthroughs in the development of messenger ribonucleic acid (mRNA) vaccines. EMA, European Medicines Agency; COVID‐19, coronavirus disease 2019; FDA, U.S. Food and Drug Administration; LNP, lipid nanoparticle

The global COVID‐19 pandemic beginning in 2020 brought mRNA vaccines into the spotlight and has greatly accelerated their deployment. Recently, the Pfizer‐BioNTech COVID‐19 vaccine became the first‐ever approved mRNA‐based vaccine available to the public, although the idea of a nucleic acid vaccine was proposed decades ago.^13^ For a long time, the susceptibility of naked RNA to extracellular RNase degradation was the major obstacle to RNA transfection; therefore, a delivery system that protected mRNA and mediated cellular internalization of mRNA was needed. The earliest solution arose as early as 1978, when scientists conducted a landmark experiment, which demonstrated that human cells could take up mRNA sequestered within the liposome and stably produce proteins from it.^14^ However, the use of mRNA as a potential therapeutic agent still remained a concept with little practicality, until in 1984, when mRNA was successfully in vitro synthesized in the laboratory.^15^ Since then, mRNA vaccine development has attracted more interest, and in 1993, Martinon and his team designed and tested the first mRNA vaccine encoding the influenza nucleoprotein (NP). They managed to elicit anti‐influenza cytotoxic T lymphocytes (CTL) responses in mice.^16^ In later years, mRNA vaccines were verified to have versatility in giving effective protection to animal hosts against various diseases, including both infectious diseases (e.g., rabies, Zika virus, and others)^17^, ^18^, ^19^, ^20^, ^21^, ^22^ and noninfectious diseases (e.g., cancer).^23^, ^24^, ^25^, ^26^, ^27^, ^28^, ^29^, ^30^, ^31^ In 2005, another revolutionary paper was published by Katalin Karikó and her colleagues, suggesting that different uridine modifications of RNA (e.g. m5C, m5U, pseudouridine Ψ, and others) could suppress the activation of different human Toll‐like receptors (TLRs), while the unmodified forms stimulated all TLRs such as TLR3, TLR7, and TLR8.^32^ This finding made a profound impact on mRNA vaccine design and further advanced the use of mRNA (Figure 1).

In this review, we provide a broad overview of the progress in mRNA vaccine technology (i.e., the engineering of mRNA sequence and in vivo delivery systems), current application and success of mRNA vaccines in the treatment of cancer, as well as a range of infectious diseases (e.g., COVID‐19).

## 2 BASICS OF mRNA VACCINES

### 2.1 mRNA biology

mRNA is single‐stranded RNA (ssRNA) encoding a genetic sequence that can be translated to a protein by ribosome.^33^ There are three main types of mRNAs that mRNA vaccines can use: conventional mRNA, self‐amplifying RNA, and circular RNA.^34^, ^35^ Conventional mRNA is a nonreplicating linear mRNA and so far has been the focus of this field. Self‐amplifying RNA (saRNA) resembles conventional mRNA, albeit containing extra elements, including 5′ and 3′ conserved sequence elements, a large open reading frame (ORF) encoding four nonstructural proteins (nsP1‐4), which form alphavirus RNA‐dependent RNA polymerase (RdRP), and a subgenomic promoter dictating the expression of gene of interest as subgenomic RNA. saRNA can achieve same efficacy with a much lower dose.^36^, ^37^, ^38^ However, reuse of saRNA may be limited by immunity against viral RdRP.^39^ Circular RNA (circRNA) can be rendered translatable by inserting an internal ribosome entry site upstream of the coding sequence. The most viable approach for generating circRNA in vitro to date utilizes the group I intron self‐splicing ribozyme, which produces large circRNA using guanosine 5′‐phosphate (GTP) and magnesium ion as cofactors.^34^, ^40^, ^41^, ^42^, ^43^ This method is also known as the permuted introns and exons method.^44^

A typical mRNA consists of a 5′ cap, a 5′ untranslated region (UTR), an ORF, a 3′ UTR, and a poly‐A tail. In the eukaryotic system, translation initiation factor 4E (eIF4E) binds to the 5′ cap, and poly‐A binding protein (PABP) binds to the 3′ poly‐A tail. Through the interaction of eIF4E and PABP with the translation initiation factor eIF4G, the mRNA circularizes, which increases its structural stability^45^, ^46^, ^47^ and allows enhanced translation. A poly‐A tail about 100 nucleotides long is preferred in mRNA design, because maintenance of a long poly(d(A/T)) stretch of DNA templates is usually difficult in the bacterial host.^48^, ^49^, ^50^ Pfizer/BioNTech utilized a poly‐A tail 110 nucleotide long with a GCAUAUGACU linker placed between the poly(A)_30_ and poly(A)_70_ sequences.^51^ Counterintuitively, recent insights into poly‐A tail biology suggested that mRNAs with shorter poly‐A tails (about 30 nt long) are generally better translated than those with longer poly‐A tails. Counteracting the roles of cytoplasmic poly(A) binding protein (PABPC) in protecting the poly‐A tail to sustain translation and in facilitating deadenylase activities to accelerate mRNA decay may be one explanation.^52^

The UTR elements impact the translational efficiency of mRNA.^53^, ^54^, ^55^ UTRs of customized mRNAs are usually adapted from highly expressed human genes. For example, UTRs of α‐globin and β‐globin were often used, and in the BNT162b2 vaccine,^56^ 3′ UTRs of hybrid AES‐mtRNR1 were adopted after screening by systematic evolution of ligands via the exponential enrichment method.^57^, ^58^, ^59^, ^60^ The ORF generally encodes a target protein antigen for the purpose of eliciting effective immune responses.

### 2.2 Innate immune responses: Something to exploit or to avoid?

Exogenous mRNAs are typically transferred to the cytosol through the endosomal pathway, along which the cargo mRNAs are readily recognized by PRRs, such as TLR‐7. The recognition triggers immediate induction of type I interferon that ultimately leads to both stimulation of innate immunity and inhibition of translation.^61^, ^62^ CureVac managed to solve this dilemma by introducing the RNActive technology,^63^ in which a mixture of naked mRNAs and protamine‐complexed mRNAs work together to activate the TLR‐7‐dependent immune response, while maintaining an acceptable level of antigen expression. The two‐component mRNA vaccine system could thus elicit balanced humoral and T cell‐mediated responses, in particular the type 1 helper T (Th1)‐biased responses.^64^, ^65^, ^66^ However, the more common practice is to avoid PRR recognition of the delivered mRNA, especially the uridine moiety, rather than to exploit it. CureVac also devised an mRNA sequence engineering method to select for the most germinal center (GC)‐rich codons, thereby reducing the presence of uridine and achieving significantly higher level of protein production.^67^ Codon optimization, which could generate misfolded proteins or even novel proteins with unknown functions, should be scrutinized on a case by case basis.^68^, ^69^, ^70^ The prevailing approach is to substitute the uridine in mRNA molecules with modified nucleosides, such as pseudouridine, N1‐methyl‐pseudouridine, or 5‐methoxyuridine.^71^, ^72^, ^73^ However, the previously unexamined issue of modified nucleosides impeding translation elongation and termination deserves due attention.^74^, ^75^

### 2.3 Manufacture of mRNA vaccines

mRNA synthesis in vitro starts from bacteriophage (usually T7) RNA polymerase‐mediated transcription of a linear DNA template. A typical final product of in vitro mRNA manufacturing is Cap 1 mRNA.^76^ The Cap 1 structure is derived from the Cap 0 structure, which has an N7‐methyl guanosine connected to the 5′ nucleotide by a 5′‐5′ triphosphate bridge. Further methylation at the 2′‐O position of Cap 0 gives the Cap 1 structure. For Cap 1 mRNA, the Cap 0 backbone enables high accessibility to the translational machinery,^77^ while both 2′‐O methylation and elimination of 5′ triphosphate contribute to the evasion of translational inhibition followed by innate immune recognition.

**FIGURE 2:**
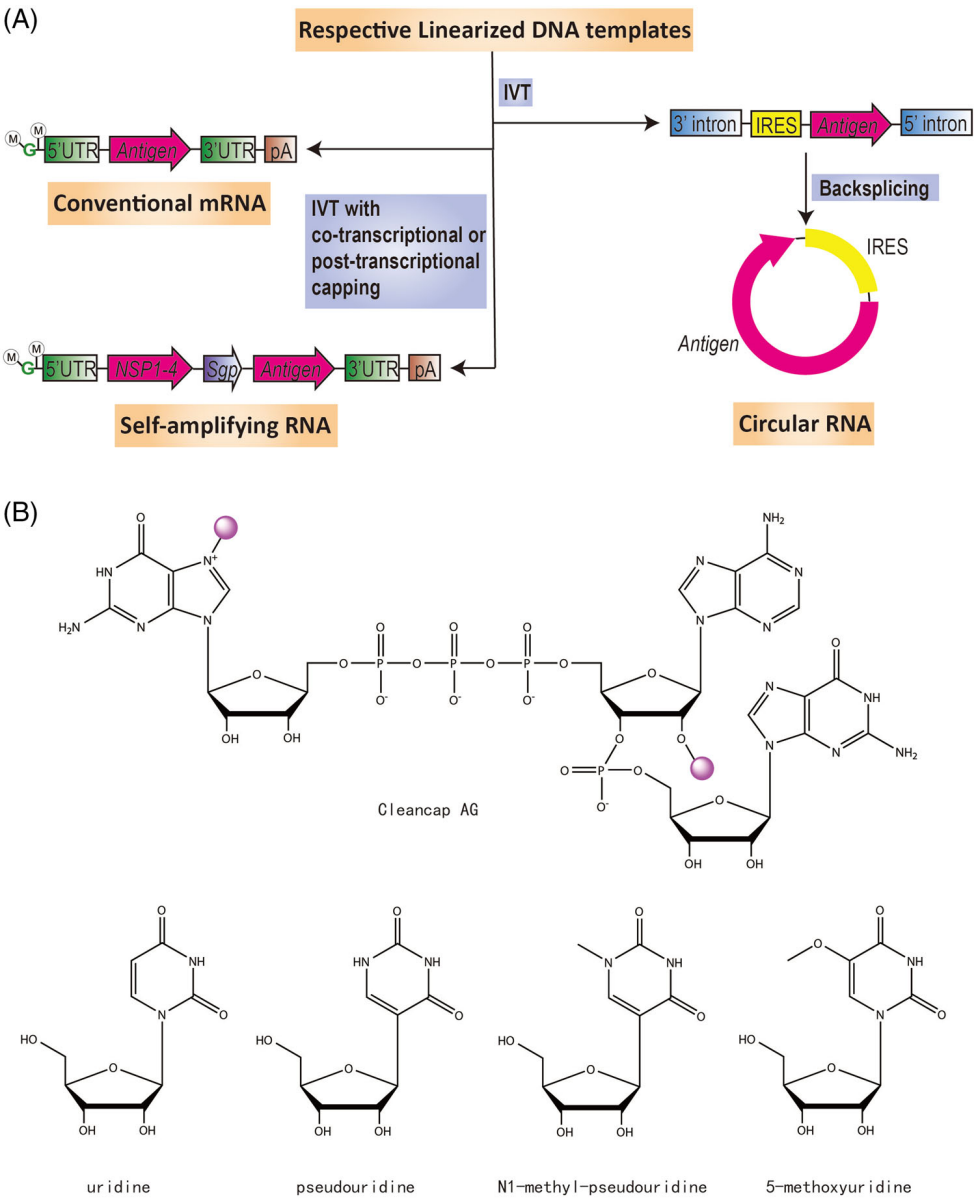
Three types of messenger ribonucleic acid (mRNA) and their production and modification. (A) Features of conventional mRNA, self‐amplifying RNA, and circular RNA, as well as their typical synthesis processes in vitro. M in circle, methyl group. G in green, guanylate. UTR, untranslated region. IVT, in vitro transcription. pA, poly (A) sequence. NSP1‐4, sequence encoding four nonstructural proteins, namely nsP1‐4, which together form RNA‐dependent RNA polymerase. Sgp, subgenomic promoter. IRES, internal ribosome entry site. (B) Structure of cap analogue Cleancap AG, as well as uridine and its modifications. Note that for Cleancap AG, the two methyl groups are highlighted with purple circles.

Capping of RNA in vitro could be done in two ways: either via co‐transcription or via post‐transcriptional enzymatic reactions. Co‐transcriptional capping is a one‐step process, a typical example of which uses CleanCap technology. This method uses a proprietary 5′ capping solution, which has a capping efficiency of 94% without the yield being significantly compromised. Enzymatic capping is a conventional two‐step process, in which vaccinia capping enzyme catalyzes the formation of Cap 0, while 2′‐O‐methyltransferase converts Cap 0 to Cap 1 structure. Co‐transcriptional capping is simple and convenient. However, CleanCap not only requires the expensive cap analogue and achieves incomplete capping but also requires alkaline phosphatase processing of uncapped RNA to avoid innate immune recognition. In addition, the DNA template must be modified to replace the GG sequence, which often appears after the TATA box of the T7 promoter, with an AG sequence. As for enzymatic capping, it is highly efficient but requires a buffer exchange between steps, which is more complicated and time‐consuming. Furthermore, certain secondary structures at the 5′ end of RNA may hamper efficient enzyme‐mediated capping (Figure 2).

These in vitro reactions can be easily scaled up for bulk manufacture of mRNA. When the reactions are complete, impurities in the reaction systems (e.g., buffers, proteins, residue substrates, DNA templates, dsRNA byproducts, and short RNA fragments) must be removed to obtain pure mRNA products. mRNA purification involves degradation of DNA templates with DNase I digestion, removal of dsRNA via cellulose chromatography, removal of small‐molecule impurities with tangential flow filtration (TFF), and removal of particulate contaminants via a sterile filter.^78^ This process is simple and convenient; however, remaining dsRNA byproducts could still trigger innate immune responses and interfere with vaccine efficacy. Strategies such as oligo dT affinity chromatography,^79^ anion exchange chromatography, and hydrogen bond chromatography^80^ can produce mRNA with even higher purity in combination with a subsequent polishing step. For oligo dT affinity chromatography, the poly‐A tail of mRNA basepairs with the oligo dT stretches under high ionic strength and dissociates under low ionic strength during elution. Other impurities that cannot bind to oligo dT are removed.^81^ For anion exchange chromatography, the net contribution of hydrogen bonding is reduced with an anion exchanger with reduced hydrogen bonding potential, and impurities like dsRNA and DNA are washed off with 1 M NaCl and 10 mM EDTA. Subsequently, ssRNA can be eluted at ambient temperature by a pH gradient. For hydrogen bond chromatography, impurities like dsRNA and DNA can also be washed off with 1 M NaCl and with 10 mM EDTA, and ssRNA can be eluted by a pyrophosphate gradient.^82^

**FIGURE 3:**
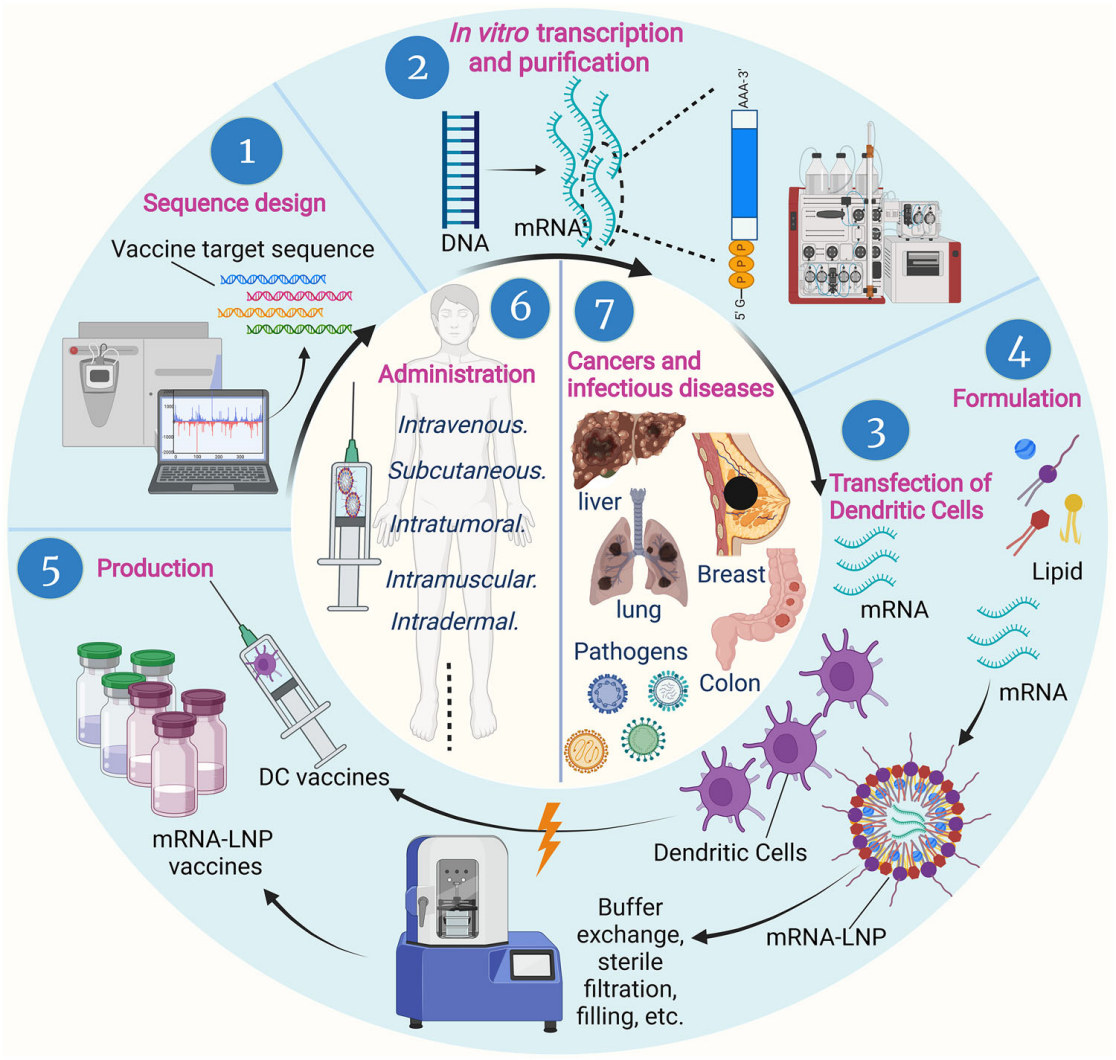
Preparation and application of messenger ribonucleic acid (mRNA) vaccines. Once pathogens or tumors are identified, sequences for the target antigens are determined by the combined efforts of sequencing, bioinformatics, and computational approaches. Target DNAs are synthesized and transcribed into mRNAs in vitro, and then mRNA transcripts are purified to remove contaminants and reactants. Purified mRNA is mixed with lipids in a microfluidic mixer to form lipid nanoparticle mRNA vaccines. Dendritic cells are loaded with candidate mRNA to form DC‐mRNA vaccines. Various vaccines are produced by scaling up, then quickly tested and stored in sterilized bottles to treat various cancers and infectious diseases through different administration methods. DC, dendritic cells; LNP, lipid nanoparticle

Complexation of mRNA with lipid nanoparticles (LNPs) can be carried out with multiple microfluidics devices placed in parallel.^83^ After purification by a TFF device and a sterile filter, the resulting mRNA‐LNPs are diluted with buffer to achieve pharmaceutical concentrations of interest (Figure 3).

### 2.4 Storage of mRNA vaccines

The two approved mRNA vaccines, mRNA‐1273 and BNT162b2, both formulated in LNPs, have shelf lives of 6 months in a frozen state at −20°C and −80 to −60°C,^84^ respectively. When thawed, they can be stored at 2–8°C for up to 1 month.^85^ These strict storage conditions challenge the global distribution of those vaccines, and many studies have been dedicated to addressing this issue.^86^, ^87^, ^88^, ^89^ Lyophilized mRNA‐LNPs achieved a stable storage duration of 6 months at 4°C and 3 months at room temperature.^88^

### 2.5 Quality control of mRNA vaccines

In 2021, the WHO released guidance on mRNA against infectious diseases, in which the drug substance (mRNA) and the drug product (final formulated vaccine) are somewhat divergent in quality control considerations.^90^ For mRNA, the identity, purity and impurities, quantification and physical state, safety attributes, reference materials, as well as stability are points of concern, while for the final formulated vaccine, the identity, purity and impurities, content, strength or quantity, safety attributes, potency, reference materials, as well as stability testing, storage, and expiry date are the focus.

### 2.6 Adverse effects of mRNA vaccines

Data from mass vaccination efforts suggest that mRNA vaccines are safe, with adverse effects that are generally acceptable in frequency and severity. Nevertheless, rapid and robust local inflammation could be induced in mice‐administrated mRNA vaccines via intradermal,^91^ intramuscular,^92^ or intranasal routes.^93^ This process was dependent on the ionizable lipid component of the LNP formulation.^94^ Myocarditis is also an adverse effect of concern, which, however, could be attributed to inadvertent intravenous injection during intramuscular administration.^88^ Furthermore, anaphylaxis could be induced by antibody response toward the polyethylene glycol (PEG) moiety, which is a rare, yet possibly fatal outcome.^95^, ^96^, ^97^ These data warrant future study to optimize the delivery system of mRNA vaccines.

### 2.7 Administration routes of mRNA vaccines

Administration method is very important for the efficacy of mRNA vaccines and can affect the organ distribution, expression kinetics, induced immune response intensity, and side effects. For prevention vaccines, such as COVID‐19 mRNA vaccines, in order to induce a strong immune response, administration is typically intramuscularly and subcutaneously (Tables 1 and 2).^98^, ^99^, ^100^ Therapeutic mRNA vaccines, such as a variety of tumor therapeutic mRNA vaccines, can be administered intravenously, intratumorally,^101^ or subcutaneously (Table 3).^102^ In addition, mRNA can be transfected into dendritic cells (DCs) in vitro to prepare a DCs cell vaccine, and the cell vaccine can be infused to prevent or treat diseases (Table 4).^103^, ^104^, ^105^

## 3 IN VIVO DELIVERY SYSTEMS FOR mRNA VACCINES

The selection of an optimal delivery system for mRNA vaccines remains one of the greatest challenges in their development. Intratumoral injection of naked mRNA can be used for cancer treatment while showing an appropriate adjuvant effect.^106^ However, due to the extremely low uptake of naked mRNA, this drug delivery system is often supplemented to reinforce the stability and efficiency of mRNA in vivo delivery. In addition, mRNA molecules are large, hydrophilic, and carry negative charges, and electrostatic repulsion makes them thermodynamically unfavorable to diffuse across the plasma membrane, which is also negatively charged.^107^, ^108^, ^109^ Once entering the body, mRNA will be further challenged by endogenous nucleases for degradation. Therefore, having a nontoxic and efficient delivery system is of critical importance to the success of mRNA vaccines. An ideal mRNA delivery system should not only enhance cellular uptake and expression of mRNA by target cells, but also protect them from nuclease degradation.^99^, ^100^, ^110^

At present, there is a large number of mRNA delivery systems being reported, many of which have already entered clinical trials. Mature delivery systems include LNP^84^, ^111^, ^112^ and protamine carriers,^113^, ^114^, ^115^ while other less popular attempts include polyethylenimine,^116^ cell‐penetrating peptides,^117^ lipopolyplexes,^118^ cationic nanoemulsions,^119^ and exosomes.^120^, ^121^ LNPs and other synthetic nanoparticles are foreign substances, which can cause related side effects ranging from mild to severe. Exosomes are naturally occurring, nano‐sized extracellular vesicles with low immunogenicity and high safety. In addition, exosomes can penetrate physiological barriers that synthetic nanoparticle carriers cannot. These characteristics make exosomes an ideal delivery carrier for mRNA.^122^ The challenges of using exosomes as carriers of mRNA delivery currently lie in large‐scale GMP‐grade production and the encapsulation efficiency of exosomes.^8^, ^123^ The three large‐scale developers of mRNA vaccines—Moderna, CureVac, and BioNTech—invested heavily in the LNP delivery system in the development of mRNA vaccines, rendering LNP the most prevalent nonviral delivery system for nucleic acid drugs.

### 3.1 LNP

**FIGURE 4:**
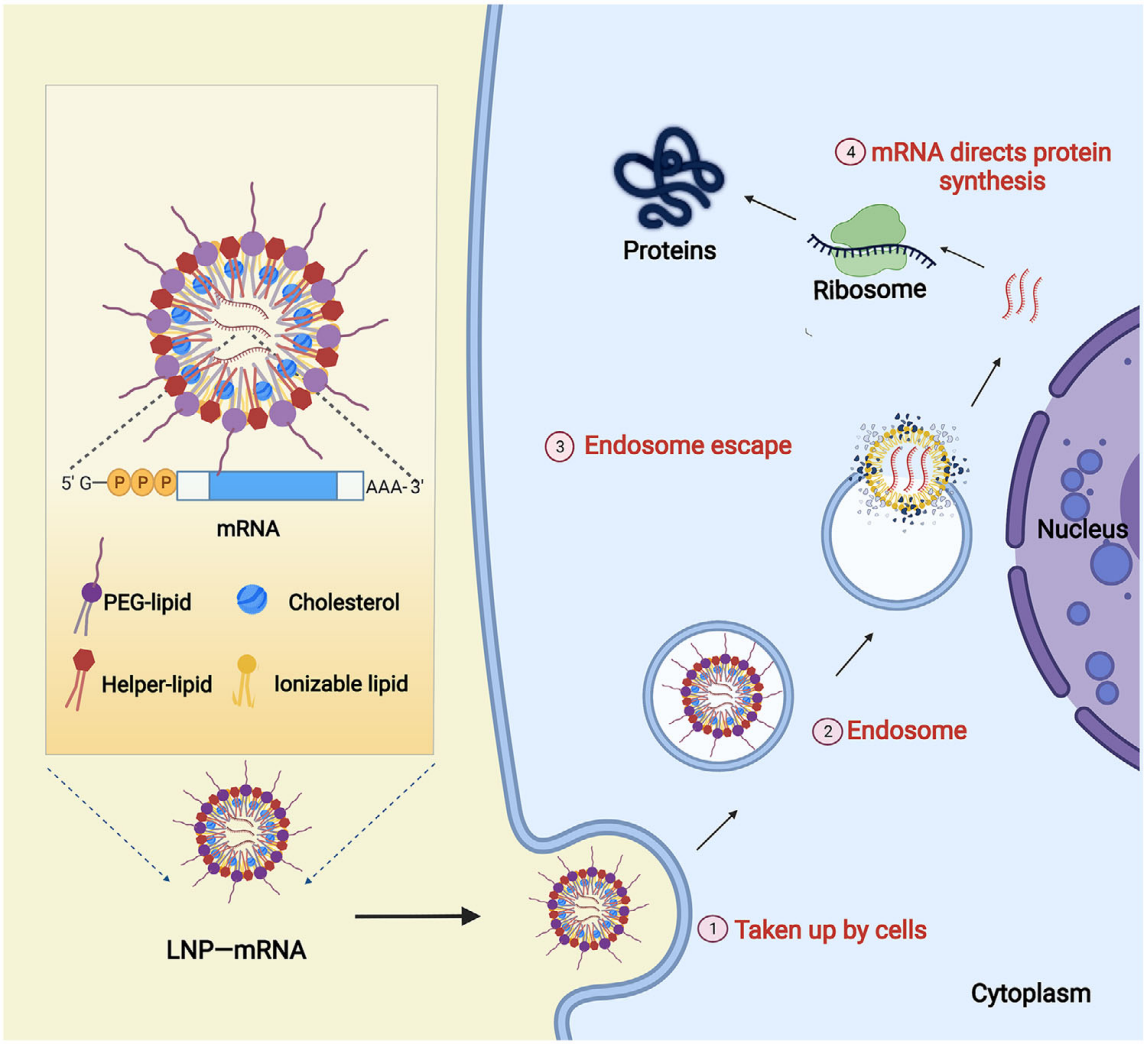
Schematic diagram of the typical structure of messenger ribonucleic acid (mRNA)‐LNP and in vivo delivery. In an acidic environment, the cationic LNP can form a complex with nucleic acids via electrostatic interaction. In the neutral environment, the formula becomes neutrally charged and thereby interacts less with serum components. Once mRNA‐LNP reaches the cell membrane, cationic phospholipids fuse with and destabilize the cell membrane, promoting the delivery of mRNA molecules. After being internalized into the cell, the mRNA‐LNP is engulfed by the endosome. The endosomal environment acidifies the ionizable phospholipids, allowing fusion with the negatively charged primary lysosomal membrane. LNP integrity is disrupted by this interaction, and therefore mRNA is released. Membrane fusions and structural changes in LNPs are thought to be the main causes of endosomal membrane destabilization and mRNA escape.

LNP is a nonviral carrier that is regarded as nontoxic and safe. The main components of LNP include PEGylated lipids, cholesterol, neutral helper lipids, and ionizable cationic lipids (Figure 4).^108^, ^124^ PEG locates on the surface of LNP. It prevents the interaction of LNP with other lipid particles or serum components,^125^ which blocks LNP from aggregating and being phagocytosed by immune cells. Cholesterol allows strong membrane fusion, which facilitates the entry of mRNA into the cytoplasm.^126^ The neutral helper lipids are, in general, saturated phospholipids.^127^ They increase the phase transition temperature of cationic liposomes, which support the formation of a lamellar lipid bilayer and stabilize its structure.^128^ The ionizable cationic phospholipids, the most critical excipient in LNP composition, determine the efficiency of mRNA delivery and transfection.^129^ They play a critical role in the intellectual property protection of the current LNP delivery systems.^130^ For example, the ionizable cationic lipids used in the Pfizer‐BioNTech and Moderna COVID‐19 mRNA vaccines (BNT162b2 and mRNA‐1273) are [(4‐hydroxybutyl)azanediyl]di(hexane‐6,1‐diyl) bis(2‐hexyldecanoate) (ALC‐0315) and SM‐102,^51^ respectively; the siRNA drug Onpattro uses Dlin‐MC3‐DMA.^131^ Microfluidic technology based on the organic solvent injection method to prepare LNPs can accurately control the size of LNPs, and this represents the most suitable preparation technology for LNPs at present.^132^

Although mRNA‐LNP vaccines have shown unprecedented potential, LNP delivery technology still has potential limitations, which hinder practical application, such as induction of allergic reactions, easy oxidative degradation, and poor preparation reproducibility.^94^, ^112^, ^133^, ^134^, ^135^, ^136^ The biggest concern is that LNP constituents may increase the risk of allergic reactions. A series of LNP‐triggered allergic reactions is currently considered to be related to several factors. First among them is the strong immunogenic nature of ionizable cationic phospholipids.^94^, ^137^ For example, after injection of the Pfizer‐BioNTech or Moderna COVID‐19 mRNA vaccines, various symptoms including swelling, pain, chills, and fever have been reported.^138^, ^139^, ^140^ These are suspected to be the consequence of the production of pro‐inflammatory cytokines such as IL‐1β and IL‐6.^141^, ^142^, ^143^ Second, an increasing number of studies has verified the immunogenicity of PEG; therefore, repeated administration of PEG can induce allergic reactions.^143^, ^144^ In addition, the hypersensitivity reactions observed after mRNA‐1273 and BNT162b2 administration are likely to be attributed to the formation of anti‐PEG antibodies after the first vaccination.^145^, ^146^ Fortunately, it has been reported that alternative lipids can be involved to prevent aggregate formation. For example, polysarcosine‐modified lipids can stabilize the LNP delivery system and prevent aggregate formation with no allergic reactions yet observed.^147^

### 3.2 Protamine

Protamine is a natural cationic polypeptide mixture and exhibits two main advantages as an mRNA delivery vehicle: first, due to its high positive charge,^114^ it can spontaneously complex with negatively charged mRNA, thereby protecting mRNA from serum nuclease degradation. Second, protamine also acts as an adjuvant.^66^, ^148^, ^149^ The protamine‐mRNA complex is recognized by immune cells via the TLR‐7/TLR‐8 pathway and activates a strong immune response to secrete type I interferon, TNF‐α, IL‐12, and other cytokines.^115^, ^150^, ^151^, ^152^, ^153^, ^154^, ^155^ Protamine‐mRNA nanoparticles of different sizes have different stimulatory effects on immune cells. For example, larger particles mainly activate monocytes and promote the production of TNF‐α, while particles smaller than 450 nm effectively stimulate plasmacytoid DCs to release IFN‐α.^152^, ^156^ To meet varying research needs, it was discovered that by adjusting the protamine to mRNA ratio and the salt concentration of the dilution solution, protamine‐mRNA can be prepared into particles with average diameters ranging from 50 to 1000 nm.^157^

CureVac rabies vaccine (CV7201) uses protamine as a delivery vehicle. CV7201 is made up of protamine and mRNA that encodes rabies virus glycoprotein (RABV‐G).^158^ In addition, CureVac also established an RNActive platform containing a protamine delivery vector. Based on RNActive,^159^ CureVac carried out clinical trials for melanoma, prostate cancer (CV9104), and nonsmall cell lung cancer (NSCLC) (CV9201 and CV9202).^160^, ^161^ In the phase IIb clinical trial of the CV9104 vaccine (NCT01817738), the overall survival (OS) of patients with metastatic castration‐resistant prostate cancer was not significantly improved compared with the placebo control group.^162^ Clinical trials for CV9201 and CV9202 also failed to show significant efficacy, which may be a result of tumor‐induced immunosuppression. Therefore, it has been proposed to combine mRNA vaccines with immune checkpoint inhibitors in future studies. Based on these results, CV9202 is undergoing a phase 1/2 study of combination immunotherapy and mRNA vaccine in subjects with NSCLC. The vaccine, administrated by intradermal injection using a needle‐free jet injector, is used in combination with the anti‐PD‐L1 antibody (Durvalumab) and/or the anti‐CTLA4 antibody (Tremlimumab) (NCT03164772).^66^

Currently, protamine‐formulated mRNA delivery systems result in limited mRNA translation efficiency and immunity strength. Compared with LNP, lower transfection efficiency of protamine may arise from its hydrophilicity, which makes it difficult to permeate the cell membrane and escape from endosome.^114^, ^163^, ^164^ Adding endosome destabilizing agents or adjusting the protamine to DOTAP (1,2‐Dioleoyl‐3‐trimethylammonium‐propane, a cationic lipids) ratio is expected to improve the transfection efficiency of protamine‐mRNA complexes.^165^

### 3.3 Tissue and cell targeted delivery systems

For mRNA therapy, it is absolutely critical that the mRNA is delivered to the right place in the body to have the desired therapeutic effect. This not only enhances the curative effect but also reduces off‐target effects. However, the liver has been found to be a natural site for accumulation upon systemic administration of mRNA‐LNP.^166^, ^167^ In order to promote the efficacy of mRNA, the delivery of mRNA to specific organs, tissues, and cells needs to be resolved.

Targeted delivery of mRNA to the spleen is beneficial for vaccines and immunotherapy because there are a large number of immune cells in this organ. It was previously reported that using intravenously‐administered positively charged RNA‐lipoplexes (RNA‐LPX) (lipid to RNA charge ratio of 5:1) targeted Luc expression predominantly in the lungs of mice. After optimally adjusting the RNA‐LPX formulation with a charge ratio of 1.3:2, which effectively targeted RNA to the spleen, LPX mediated mRNA efficient uptake and expression of the encoded antigen by DC populations and macrophages.^168^

Selective organ targeting (SORT) nanoparticles are in the development for mRNA delivery to nonliver tissues. LNPs are usually composed of ionizable cationic lipids, amphiphilic phospholipids, cholesterol, and poly(ethylene glycol) lipids. SORT molecules are the fifth component added to the traditional four‐component LNPs, with the added SORT molecules controlling biodistribution, global/apparent pKa, and serum protein interactions of the SORT nanoparticles. With the help of SORT molecules, LNPs can specifically target the lungs, the spleen, and the liver, and effect mRNA delivery and gene editing of related cells, such as epithelial cells, endothelial cells, B cells, T cells, and hepatocytes.^41^, ^169^

## 4 mRNA VACCINES FOR PREVENTION OF INFECTIOUS DISEASES

Infectious diseases are transmissible diseases caused by infection by pathogens, such as bacteria and viruses. The first active defense of host against those pathogens is innate immunity, which is usually evoked by PRR recognition of pathogen‐associated molecular patterns (PAMPs). PAMPs are a series of conserved and highly abundant molecular motifs within a class of pathogens. Innate immunity utilizes various mechanisms to combat invading pathogens, such as inflammation, engulfment, and the complement system. Later, adaptive immunity, elicited by antigen presentation in an inflammatory milieu, reacts specifically to antigens of pathogens by secreting antibodies and activating cytotoxic lymphocytes. Effective innate immunity creates the optimal microenvironment to foster a potent adaptive immunity, or it will otherwise be too weak and too late to prevail over pathogens in cases of serious infections. However, prophylactic vaccination is able to boost a strong adaptive defense and adaptive memory before infection takes place, achieving enduring immunity against relevant pathogens. mRNA vaccines encoding pathogen antigens are an active family of vaccines for controlling infectious diseases.^1^, ^170^

### 4.1 COVID‐19 mRNA vaccines

The COVID‐19 pandemic caused by the severe acute respiratory syndrome coronavirus 2 (SARS‐CoV‐2) has more than 447 million cases and six million deaths globally as of March 8, 2022. It has devastating effects on health, as well as social and economic situations. Two mRNA vaccines were released after a short period of development. BNT162b2, under the brand name Comirnaty, and mRNA‐1273, under the brand name Spikevax, were developed by Pfizer–BioNTech and Moderna, respectively. They were authorized for emergency use in December 2020 and have since made considerable contributions to infection control.

#### 4.1.1 Design of the COVID‐19 mRNA vaccines

The two approved COVID‐19 mRNA vaccines, BNT162b2 and mRNA‐1273 (see Table 1),^171^ both contain mRNAs encoding the SARS‐CoV‐2 spike (S) protein, a 1273 amino acid long type I transmembrane glycoprotein that dictates the infectivity of the virus. One common feature is that they both harbor two substitutions of amino acids, namely K986P and V987P, in the peptide chain to stabilize the prefusion conformation of the S protein.^112^, ^172^ In addition, they both use N1‐methyl‐pseudouridine, instead of uridine, as a substrate to limit innate immune responses to mRNAs. However, the capping strategies differ. Capping for BNT162b2 is fulfilled co‐transcriptionally with the previously described CleanCap cap analogues,^84^, ^173^ while capping for mRNA‐1273 is achieved by post‐transcription reactions catalyzed by vaccinia capping enzyme and 2′ O‐methyltransferase as discussed above.^84^, ^174^

**TABLE 1:** Information on COVID19 mRNA vaccines approved for use

Category	Pfizer‐BioNTech	Moderna
Name product	BNT162b2	mRNA‐1273
Lipid nanoparticle components and ratio	ALC‐0315/DSPC/Cholesterol/ALC‐0159 = 46.3:9.4:42.7:1.6	SM‐102/DSPC/Cholesterol/PEG2000‐DMG = 50:10:38.5:1.5
Ionizable nitrogen/phosphate molar raio	6	Estimated to be 6
Excipients	KH2PO4; Na2HPO4; KCl; NaCl; Sucrose; Water for injection	Tris; sodium acetate; sucrose; water for injection
Ages recommended	5+ years old	18+ years old
mRNA dose; route of administration	30 μg; intramuscular	100 μg; intramuscular
Primary series	Two doses; given 3 weeks apart	two doses; given 4 weeks apart
Booster dose	Everyone aged 18 years and older should get a booster dose of either Pfizer‐BioNTech or Moderna (COVID‐19 vaccines) 5 months after the last dose in their primary series.	Everyone aged 18 years and older should get a booster dose of either Pfizer‐BioNTech or Moderna (COVID‐19 vaccines) 5 months after the last dose in their primary series.
	Teens 12–17 years old should get a Pfizer‐BioNTech COVID‐19 Vaccine booster 5 months after the last dose in their primary series.	Null
When fully vaccinated	2 weeks after 2nd dose	2 weeks after 2nd dose

*Note*: The table's data adapted from https://www.sciencedirect.com/science/article/pii/S0378517321003914 and https://www.cdc.gov/coronavirus/2019‐ncov/vaccines/different‐vaccines.html.

Abbreviation: ALC‐0315, ([(4‐hydroxybutyl)azanediyl]di(hexane‐6,1‐diyl) bis(2‐hexyldecanoate)); ALC‐0159, 2‐[(polyethylene glycol)‐2000]‐N,N‐ditetradecylacetamide; COVID‐19, corona virus disease‐2019; DSPC, 1,2‐Distearoyl‐sn‐glycero‐3‐phosphocholine; mRNA, messenger ribonucleic acid; PEG, polyethylene glycol.

Other modifications of the S antigen were also investigated. In one study, wild‐type S antigen versions with two proline substitutions (2P), four additional proline substitutions (6P), furin cleavage site elimination, and endoplasmic reticulum retention signal elimination were compared for their capacity to induce neutralizing antibodies. The S antigen with both 2P and furin cleavage site elimination outperformed the others, inducing Th1‐biased responses in both the mouse and nonhuman primate models.^175^ In another study, glycosylation site depletion in the receptor‐binding domain or the S2 domain of the S antigen exposed more conserved epitopes, and the mRNA vaccine encoding this type of S protein conferred broad protection against wild‐type virus and different variants of concern. The resulting misfolded protein product induced cell apoptosis and potent T cell responses.^176^

Although the S antigen is the predominant target for COVID‐19 mRNA vaccines, other structural and nonstructural genes have been proposed as targets.^177^, ^178^, ^179^, ^180^, ^181^, ^182^, ^183^, ^184^ These genes are not under constant high selection pressure and are thus more conserved. Structural gene encoding NP (N), which is indispensable in the viral life cycle, was shown to elicit protective immune responses.^177^, ^180^ Nonstructural genes, although not present in viral particles, can also be exploited to mark the infected cells for destruction. Studies have suggested that nonstructural protein 3 (Nsp3), Nsp8, and Proteinase 3CL‐PRO could serve as potential targets.^181^, ^182^, ^183^

#### 4.1.2 Mechanisms of action of COVID‐19 mRNA vaccines in humans

mRNA vaccines can induce adaptive immunity against specific antigens. In the case of COVID‐19 mRNA vaccines, while the induction of neutralizing antibodies, which inactivate the live viruses, is currently a major concern, the roles of T cell immunity in the process are becoming more appreciated.^185^

Analyses on vaccinated individuals revealed that, after the first dose, COVID‐19 mRNA vaccines elicited rapid Th1 and follicular helper T (Tfh) cell responses, which positively correlated with the level of follow‐up CD8^+^ T cells and neutralizing antibodies after the second dose. Importantly, CD4^+^ and CD8^+^ T cells generated by mRNA vaccination exhibited memory characteristics, which could be pivotal to recall responses for future infection.^4^, ^186^ In another study, by directly probing GC responses in the lymph nodes (LNs) of vaccinees, a connection between GC formation and the generation of neutralizing antibodies and memory B cells after COVID‐19 mRNA vaccination was proposed.^187^ It was demonstrated in a recent study that, after COVID‐19 mRNA vaccination, GCs in LNs were robustly developed, and vaccine mRNA and the resulting S antigen were present in the GCs for a prolonged period of time. Nine months after vaccination, the titre of spike‐specific IgG dropped to about 1/20 of the peak level; however, a third‐dose booster managed to generate another new peak of antibody titer within 1 week.^188^ Several other studies also corroborated a waning of immune responses against COVID‐19 over time, arguing for the necessity of further boosters to consolidate long‐term immunity.^19^, ^189^, ^190^, ^191^, ^192^, ^193^

Mucosal immunity, which directly responds to viral challenges, is also a concern. Disappointingly, in contrast to systemic immunity, mucosal immunity was poorly activated following standard intramuscular administration.^194^ In light of this, a mucosal booster strategy was proposed to elevate mucosal immunity against COVID‐19.^195^

#### 4.1.3 Real‐world efficacy and strategies of COVID‐19 mRNA vaccines

Data from real‐world observational studies have been accumulating, as mass vaccination programs have been implemented worldwide.^196^ A general study on mRNA vaccines based on health care staff, first responders, and other frontline workers demonstrated vaccine effectiveness of 91% for those who received two doses, and 81% for those who had only 1 dose. Moreover, for those who contracted the virus, the vaccinated group benefited by experiencing a 40% reduction in viral load, a 58% lower risk of febrile symptoms, and 2.3 days less sickbed time.^197^

In one study conducted by Israel's largest health care organization on the BNT162b2 vaccine, by exploring the data collected at days 14 through 20 after the first dose and at day 7 or more days after the second dose, the vaccine had effectiveness rates of 46% and 92% for documented infection, 57% and 94% for symptomatic COVID‐19, 74% and 87% for hospitalization, and 62% and 92% for severe disease. Importantly, estimated mortality avoidance rate was 72% at days 14 through 20 after the first dose.^198^ Another study was carried out in a large health maintenance organization in Israel, with individuals who frequently underwent polymerase chain reaction testing for SARS‐CoV‐2 infection. For the prevention of asymptomatic SARS‐CoV‐2 infection, the BNT162b2 vaccine showed an estimated effectiveness of 89% 7 days after two doses, in contrast to 61% 2 weeks after 1 dose.^199^ A third study involved health‐care workers in England, who also underwent regular asymptomatic testing. The effectiveness of BNT162b2 vaccine was 70% 21 days after the first dose and 85% 7 days after the second dose.^200^ A more recent study confirmed the effectiveness of the BNT162b2 vaccine in a large health provider cohort in Israel, achieving a protection rate of 90% and 94% against SARS‐Cov‐2 infection and COVID‐19 after two doses, respectively. Results from immunosuppressed patients were also impressive, reaching 71% protection against infection.^201^

Recently published results for the two‐dose mRNA‐1273 vaccines were also encouraging. mRNA‐1273 provided protection against COVID‐19 infection (87.4%), COVID‐19 hospitalization (95.8%), and hospital mortality (97.9 %). Effectiveness against symptomatic COVID‐19 was 88.3%, compared to 72.7% against asymptomatic cases. A moderate effectiveness of 8.2–33.6% was observed among individuals with COVID‐19 history, suggesting the need for vaccination of those who have recovered from the disease.^202^

To date, there have been five SARS‐CoV‐2 variants of concern: alpha (B.1.1.7 and descendant lineages), beta (B.1.351), gamma (P.1), delta (B.1.617.2 and AY lineages), and omicron (B.1.1.529 and BA lineages), with each causing a surge in cases and mortalities.^203^ Reduced neutralizing activity by vaccines against these variants was observed, most notably for the latest variants, Delta and Omicron.^204^, ^205^, ^206^, ^207^ Fortunately, strategies such as sequential immunization and additional boosters have shown promise for limiting the breakthrough infection, hospitalization, severe disease, and death caused by SARS‐CoV‐2.^26^, ^208^, ^209^, ^210^, ^211^ In addition, there are also suggestions for alternative strategies, such as constantly updating vaccines with newly prevalent variant sequences, polyvalent vaccines, or pan‐coronavirus strategies.^212^

A recent real‐world study in Qatar revealed that two doses of an mRNA vaccine achieved peak protection rates of 41.6% and 51.7% against symptomatic infection by Omicron subvariants BA.1 and BA.2 3 months after the last dose, before dropping to 10% or lower.^196^ Similar waning protection was also observed for the third dose, where moderate peak protection rates of 59.9% and 43.7% against infection by the two subvariants, respectively, were achieved 1 month after the last dose. Encouragingly, two doses of an mRNA vaccine reduced COVID‐19 hospitalization and death by 70%–80%, and a third dose further reduced the rate by >90%.^196^

These data establish that the COVID‐19 mRNA vaccines currently in use are substantially efficacious. Nevertheless, further improvements are needed to combat the constantly evolving virus.

### 4.2 mRNA vaccines for other infectious diseases

Clinical trials of multiple mRNA vaccines targeting other infectious diseases, such as influenza,^175^, ^213^ Zika virus,^18^, ^22^, ^214^ and rabies^215^, ^216^ are underway. However, the development of these vaccines is not comparable to the rate of progress that development of the SARS‐CoV‐2 vaccine has achieved (Table 2).

**TABLE 2:** Clinical trials of mRNA vaccines targeting other infectious diseases

Funding source	Vaccine name	Target	Route	Phase	NCT Number
Moderna	mRNA‐1189	Epstein‐Barr Virus Infection	Intramuscular	Phase I	NCT05164094
	mRNA‐1345	Respiratory Syncytial Virus (RSV)	Intramuscular	Phase II/III	NCT05127434
	mRNA‐1647	Cytomegalovirus Infection	Intramuscular	Phase III	NCT05085366
	mRNA‐1010	Seasonal Influenza	Intramuscular	Phase I/II	NCT04956575
	mRNA‐1893	Zika Virus	Intramuscular	Phase II	NCT04917861
	mRNA‐1443	CMV	Intramuscular	Phase I	NCT03382405
	mRNA‐1325	Zika	Intramuscular	Phase I	NCT03014089
	mRNA‐1653	hMPV/PIV3	Intramuscular	Phase I	NCT04144348; NCT03392389
	mRNA‐1851 (VAL‐339851)	Influenza A (H7N9)	Intramuscular	Phase 1	NCT03345043
	mRNA‐1440 (VAL‐506440)	Influenza A (H10N8)	Intramuscular	Phase 1	NCT03076385
	mRNA‐1010	Influenza A (H1N1, H3N2), influenza B (Yamagata lineage, Victoria lineage)	Intramuscular	Phase I/II	NCT04956575
	mRNA‐1944	Chikungunya	Intramuscular	Phase I	NCT03829384
	mRNA‐1388 (VAL‐181388)	Chikungunya	Intramuscular	Phase I	NCT03325075
CureVac	CV7201	Rabies	Intradermal, intramuscular	Phase I	NCT02241135
	CV7202	Rabies	Intramuscular	Phase I	NCT03713086
CureVac AG	CVSQIV	Influenza	Intramuscular	Phase I	NCT05252338
GSK	GSK3903133A	Rabies	Intramuscular	Phase I	NCT04062669
NIAID	BG505 MD39.3 mRNA; BG505 MD39.3 gp151 mRNA; BG505 MD39.3 gp151 CD4KO mRNA	HIV Infections	Intramuscular	Phase I	NCT05217641

*Note*: The table summarizes the clinical trials of mRNA vaccines targeting other infectious diseases registered at Clinical Trials.gov.

Abbreviations: CMV, cytomegalo virus; GSC, Glaxosmithkline Plc; mRNA, messenger ribonucleic acid; NIAID, National Institute of Allergy and Infectious Diseases.

## 5 mRNA CANCER VACCINES

Although the COVID‐19 pandemic has accelerated the application of mRNA vaccines against viral infectious diseases, mRNA cancer vaccines were the earliest direction assessed in exploratory clinical trials, and remain a field with the most intense competition and the most concentrated R&D pipelines.^217^

**FIGURE 5:**
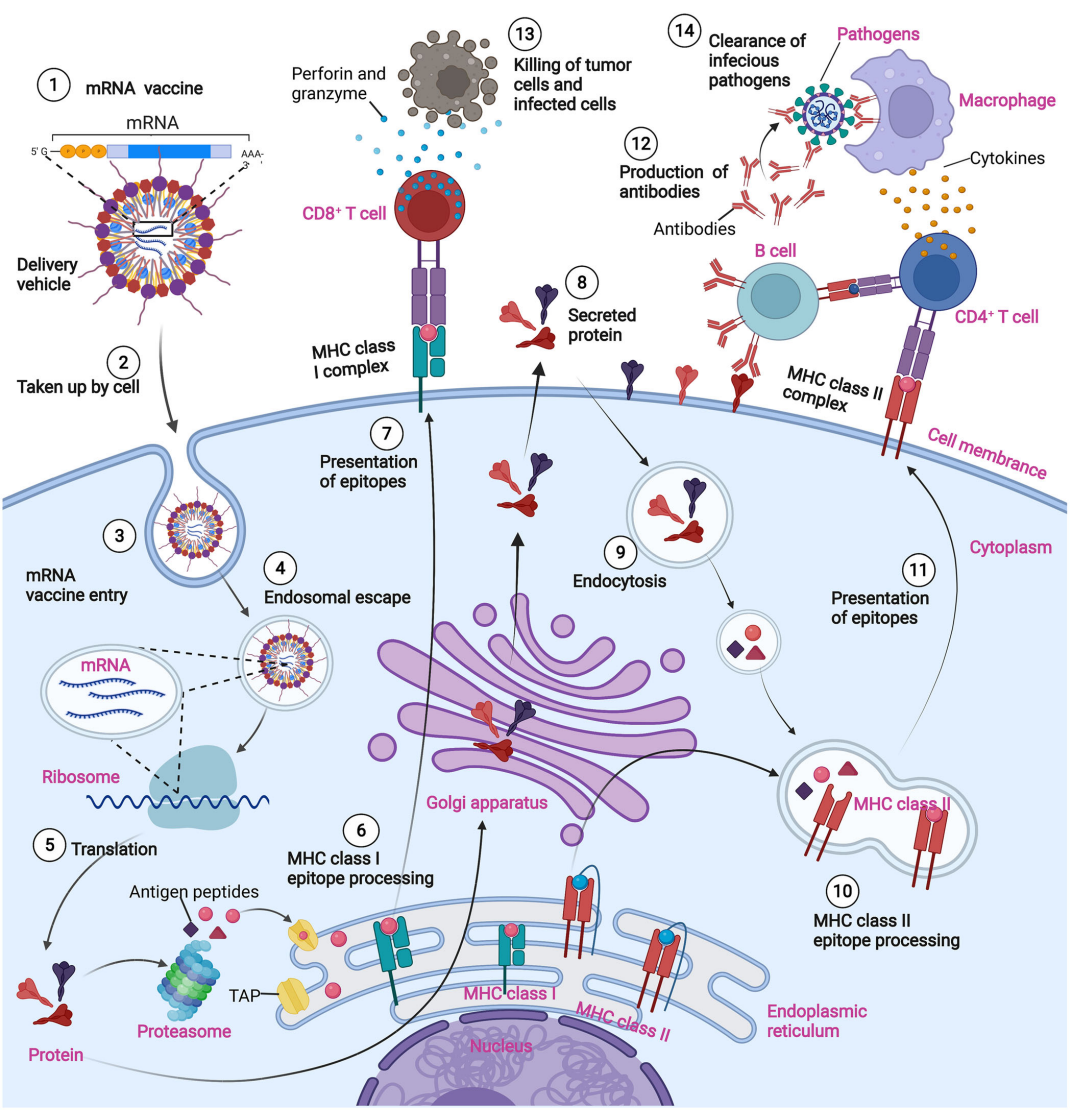
Mechanisms of messenger ribonucleic acid (mRNA) vaccines for infectious diseases and cancers. mRNA molecules encoding tumor antigens are injected into body (either with or without delivery vehicles). The mRNA molecules are taken up and translated into protein antigens by antigen presenting cells (APCs). After proteasomal processing of proteins, antigen peptides associate with major histocompatibility complex (MHC) Class I molecule in the endoplasmic reticulum and are transferred to the APC surface, activating CD8+ T cells for a specific cellular immune response. Protein antigens, which are sorted for the endosome route, can activate CD4+ T cells via the MHC Class II presentation pathway. The secretory protein antigen or membrane antigen encoded by mRNA can stimulate B cells to produce neutralizing antibodies, and activate phagocytes such as macrophages to secrete inflammatory cytokines, facilitating the clearance of circulating infectious pathogens and tumor cells

There are two types of cancer vaccines with fundamentally different working principles: prevention vaccines and therapeutic vaccines (Figure 5). It is estimated that 15% of all human cancers are associated with viral infection (e.g., human papillomavirus, hepatitis B virus, and others).^218^, ^219^, ^220^, ^221^, ^222^ Therefore, vaccines that protect against certain viruses serve as prophylactic measures against certain cancers. In contrast, therapeutic vaccines address existing cancers. The mRNA‐based vaccine allows the use of vaccines as an available immunotherapy for cancer and other nonviral diseases. Most cancer vaccines fall into the therapeutic category. In general, they target tumor‐associated antigens (TAA), which are preferentially expressed in cancerous cells.^8^

Currently, mRNA tumor vaccines are applied in two ways. One directly delivers mRNA into the body using delivery vehicles such as LNP or protamine,^223^, ^224^, ^225^ and the other ex vivo transfects DCs with tumor antigen‐encoding mRNA to prepare a DC vaccine.^226^, ^227^ In addition, although mRNA encoded immunostimulants are not strictly part of tumor vaccines, their ability to turn cold tumors, with no or low immune response, into well‐responsive hot tumors has attracted attention. Consequently, many clinical trials have attempted to enhance antitumor efficacy^228^, ^229^, ^230^ (Table 3).

**TABLE 3:** Currently clinical trials with mRNA tumor vaccines

Vaccine type	NCT Number	Cancer type	Status	Phases	mRNA	Combo	Route	Completion
mRNA encoding TAAs	NCT02410733	Melanoma	Active, not recruiting	I	TAAs: NY‐ESO‐1/MAGE C3/ tyrosinase/TPTE; Lipo‐MERIT	Null	i.v.	2023/5
	NCT01890213	Stage III colon cancer	Completed	I	An alphavirus replicon (VRP) encoding the protein (CEA); AVX701	Null	i.m.	2019/7
	NCT04534205	Unresectable/Metastatic/Recurrent) HNSCC	Recruiting	II	TAAs:E6/E7; BNT113	Pembrolizumab (i.v. infusion)	i.v.	2025/5
	NCT04526899	Melanoma stage III, IV; unresectable melanoma	Recruiting	II	TAAs:NY‐ESO‐1, MAGE‐A3, tyrosinase, TPTE; BNT111	Cemiplimab (i.v. infusion)	i.v.	2023/12
	NCT04382898	Prostate cancer	Recruiting	I/II	TAAs: RBL038/RBL039/RBL‐040/RBL‐041/RBL‐045; BNT‐112	Cemiplimab (i.v. infusion)	i.v.	2023/7
	NCT00204516	Malignant melanoma	Completed	I/II	TAAs: Melan‐A/ Mage‐A1/Mage‐A3/Survivin/GP100 /Tyrosinase; mRNA coding for melanoma‐associated antigens	GM‐CSF (s.c.)	s.c.	2012/7
	NCT03164772	Metastatic NSCLC; NSCLC	Completed	I/II	TAAs:NY‐ESO‐1/MAGE‐C2/MAGE‐C1/survivin/5T4/MUC1; BI 1361849	Durvalumab + tremelimumab	Null	2021/10/29
mRNA encoding neoantigens	NCT03289962	Melanoma; NSCLC; bladder cancer; COAD; TNBC	Active, not recruiting	I	Neo‐Ag (mRNA); autogene cevumeran	Atezolizumab (i.v. infusion)	i.v.	2024/2/1
	NCT03313778	Solid tumors	Recruiting	I	Neo‐Ag (mRNA); mRNA‐4157	Pembrolizumab (i.m. injection)	i.m.	2022/6/30
	NCT04161755	Pancreatic cancer	Active, not recruiting	I	Neo‐Ag (mRNA); RO7198457	Atezolizumab + mFOLFIRINOX	Null	2023/11/11
	NCT03948763	Pancreatic neoplasms; colorectal neoplasms; carcinoma, NSCLC	Active, not recruiting	I	KRAS mutations: G12D/G12V/G13D/G12C; mRNA‐5671/V941	Pembrolizumab (i.v. infusion)	i.m.	2022/8/12
	NCT04397926	NSCLC	Recruiting	I	Neo‐Ag (mRNA)	Null	s.c.	2022/5
	NCT04487093	NSCLC	Recruiting	I	Neo‐Ag (mRNA)	EGFR‐TKI+ anti‐angioge	s.c.	2022/12/1
	NCT02035956	Melanoma	Completed	I	Neo‐Ag (mRNA):IVAC MUTANOME; NY‐ESO‐1/tyrosinase/personalized neoantigen petide	RBL001/RBL002	i.n.	2019/10
	NCT03597282	Metastatic Melanoma	Terminated	I	Neo‐Ag (mRNA):NEO‐PV‐01	Nivolumab (i.v.) + Adjuvant + APX005M + ipilimumab	s.c.	2020/8/11
	NCT03166254	NSCLC	Withdrawn	I	Neo‐Ag (mRNA):NEO‐PV‐01	Pembrolizumab + Poly ICLC	s.c.	2027/5
	NCT03468244	Advanced ESC; GAC; PAAD; COAD	Recruiting	I	Neo‐Ag (mRNA)	Null	s.c.	2021/12/31
	NCT02316457	TNBC	Active, not yet recruiting	I	IVAC_W_bre1_uID/IVAC_M_uID	Null	Null	2023/12
mRNA encoding neoantigens	NCT04163094	Ovarian Cancer	Recruiting	I	W_ova1	(Neo‐)Adjuvant Chemotherapy	i.v.	2023/12
	NCT03380871	Carcinoma, NSCLC; Nonsquamous NSCLC	Completed	I	Neo‐Ag (mRNA):NEO‐PV‐01	Pembrolizumab + Adjuvant + Carboplatin + Pemetrexed	s.c.	2021/2/5
	NCT02897765	UBC; Bladder Tumors; TCC of the Bladder; Melanoma; Skin Cancer; NSCLC	Completed	I	Neo‐Ag (mRNA):NEO‐PV‐01	Adjuvant + Nivolumab (i.v.)	s.c.	2020/5
	NCT03815058	Advanced Melanoma	Active, not recruiting	II	Neo‐Ag (mRNA); RO7198457	Pembrolizumab (i.v. infusion)	i.v.	2022/9/1
	NCT03897881	Melanoma	Active, not recruiting	II	Neo‐Ag (mRNA); mRNA‐4157	Pembrolizumab (i.v. infusion)	Null	2024/6/30
	NCT04267237	NSCLC	Withdrawn	II	Neo‐Ag (mRNA); RO7198457	Atezolizumab	i.v.	2025/9/30
	NCT04486378	Colorectal Cancer Stage II/III	Recruiting	II	Neo‐Ag (mRNA); RO7198457	Null	i.v.	2027/7
	NCT03480152	Melanoma; COAD; GAC; genitourinary cancer; LIHC	Terminated	I/II	Neo‐Ag (mRNA)	Null	i.m.	2019/11/5
	NCT03908671	Esophageal cancer; NSCLC	Not yet recruiting	NA	Neo‐Ag (mRNA)	Null	s.c.	2022/9
mRNA encoding immunostimulants	NCT03788083	Breast cancer; early‐stage breast cancer	Recruiting	I	Trimix mRNA; mRNA encoding CD40L, CD70, acTLR4	Null	i.t.	2022/12/30
	NCT03394937	Melanoma	Recruiting	I	ECI‐006;mRNA encoding tyrosinase, gp100, MAGE‐A3, MAGE‐C2, PRAME	Null	i.n.	2021/1/29
	NCT03291002	Melanoma (Skin); SCC; HNSCC; ADCC	Active, not yet recruiting	I	CV8102; mRNA encoding TLR‐7,8 and RIG‐1	anti‐PD‐1 therapy	Null	2023/2
	NCT03946800	Solid Tumors	Recruiting	I	MEDI1191; mRNA encoding IL‐12	Durvalumab (i.v.)	i.t.	2027/1/15
	NCT03739931	TNBC; HNSCC; NHL; Melanoma; NSCLC	Recruiting	I	mRNA‐2752; mRNA encoding OX40L, IL‐23, IL36γ	Durvalumab	i.t.	2023/1/30
	NCT03871348	Metastatic neoplasm	Recruiting	I	SAR441000; mRNA encoding IL‐12sc, IL‐15sushi, IFN α, GM‐CSF	Cemiplimab REGN2810 (i.v.)	i.t.	2024/4
	NCT03323398	Solid tumor; ovarian cancer	Recruiting	I/II	mRNA‐2416; mRNA encoding OX40L	Durvalumab (i.v.)	i.t.	2022/9/20
	NCT04455620	Solid tumor	Recruiting	I/II	BNT151; mRNA encoding IL‐2	Null	i.v.	2025/1/1

*Note*: The table summarizes the currently clinical trials with mRNA tumor vaccines registered at *Clinical Trials.gov*.

Abbreviations: ADCC, adenoid cystic arcinoma; CD40L, CD40 ligand; CEA, carcinoembryonic antigen; COAD, colon adenocarcinoma; ESC, esophageal squamous carcinoma; GAC, gastric adenocarcinoma; HNSCC, head and neck squamous cell carcinoma; i.m., intramuscular injection; i.n., intranodal injections; i.t., intratumoral injection; i.v., intravenous injection; LIHC, liver hepatocellular carcinoma; LNP, Lipid nanoparticles; mRNA, messenger ribonucleic acid; NA, not available; Neo‐Ag, neoantigen; NHL, non‐Hodgkin's lymphoma; NSCLC, non‐small cell lung cancer; OX40L, OX40 ligand; PAAD, pancreatic adenocarcinoma; PD‐L1, programmed death ligand‐1; RIG‐1, retinoic acid induced genes 1; s.c.,subcutaneous injection; SCC, squamous cell carcinoma; STES, stomach and esophageal carcinoma; TAAs, tumor‐associated antigens; TCC, transitional cell carcinoma of the bladder; TNBC, triple negative breast cancer; UBC, urinary bladder cancer.

The development of a therapeutic cancer vaccine is a research focus for many: using “cancer vaccine” as keywords in ClinicalTrials.gov results in more than 2000 clinical registered projects; however, this goal remains extremely challenging. After huge investments of human and financial resources, many blockbusting cancer vaccines failed in phase III trials, such as Stimuvax (Merck, USA), which targets the tumor antigen mucin (MUC1),^231^ and the lead product of GlaxoSmithKline, GSK1572932A,^232^ which targets melanoma‐related antigen 3 (MAGE‐A3).^233^, ^234^ These vaccines target a single TAA; however, for the development of mRNA tumor vaccines, simultaneous targeting of multiple TAAs or neoantigens is more promising (Table 3).

### 5.1 mRNA vaccines simultaneously encoding multiple TAAs

Simultaneous targeting of multiple TAAs reduces the chance of tumor antigen escape due to mutations and low expression of target antigens and thus increases the robustness and precision of specific antitumor responses.^235^, ^236^, ^237^ The majority of ongoing clinical trials employ this strategy. More than 90% of melanoma patients express at least one of the four prevalent TAAs: NY‐ESO‐1, MAGE‐A3, Tyrosinase, and TPTE.^8^, ^238^ BNT111 is a lead candidate from the BioNTech FixVAC platform, which is experiencing the fastest research and developmental progress, and encodes all four TAAs of melanoma. According to a completed phase I clinical trial (NCT02410733), most melanoma patients given this vaccine that showed stable disease (SD) and partial responses have achieved ongoing survival. Some patients have survived for more than 2 years with TAA‐specific T cells still remaining at a detectable level in their body.^239^ Based on the safety and preliminary efficacy of BNT111 demonstrated in that phase I trial, a phase II clinical trial of BNT111 in combination with Libtayo (Cemiplimab) was initiated in May 2021(NCT04526899), which targets patients with unresectable anti‐PD1‐refractory/relapsed melanoma.^240^

More DNA mutations can produce more candidate peptides and lead to an increase in tumor antigens that can be presented. Several studies have demonstrated that tumor patients with a high tumor mutational burden are often associated with stronger T cell response and better clinical outcomes.^241^, ^242^ Therefore, multitargeting mRNA vaccines have shown promising prospects in the treatment of highly immunogenic melanomas but have not progressed as well in other tumor types.^243^, ^244^, ^245^ For example, the mRNA vaccine BI‐1361849 (formerly called CV9202), developed by Curevac, is currently undergoing phase I/II clinical trials for treatment of NSCLC. BI‐1361849 encodes six TAAs (NY‐ESO‐1, MAGE‐C2, MAGE‐C1, survivin, 5T4, and MUC1). The completed phase I clinical trial (NCT01915524) showed that BI‐1361849 was well tolerated and immunogenic.^66^ Furthermore, a phase I/II clinical trial (NCT03164772) of BI‐1361849 in combination with durvalumab and tremelimumab for NSCLC has also been completed. It is speculated that this may be related to poor clinical efficacy. CureVac terminated its cooperation agreement with Germany's Boehringer‐Ingelheim CV9202 project for the treatment of NSCLC in June 2021.^246^

Although current studies focus on aberrantly or overexpressed autoantigens in tumors, several obstacles constrain the further clinical application of TAA‐targeted vaccines. First, only a limited number of TAAs have been identified for certain solid tumors, and extensive mutations in TAAs lead to immune escape and drug resistance. Second, TAAs exhibit poor immune specificity and immunogenicity, because they exist in normal tissues. Additionally, some TAAs used to be embryonic antigens, which have already induced an immune tolerance during the individual developmental processes. Lastly, TAA‐targeted therapy may trigger autoimmune responses can lead to serious safety concerns.^247^, ^248^ Therefore, developing cancer vaccines based on tumor‐specific antigens with high immunogenicity has been a hope of oncologists. mRNA tumor vaccines that encode neoantigens provide a new research path.^27^, ^249^, ^250^, ^251^

### 5.2 Personalized neoantigen‐encoding mRNA vaccines

Malignant cells are characterized by continuous and rapid proliferation, with the expansion of the tumor population often accompanied by mutations in a large number of genes, generating multiple neoantigens. Neoantigens originate from random somatic mutations in tumor cells and do not exist in normal cells. Therefore, neoantigens are recognized as nonself by the immune system and are presented to T cells by human leukocyte antigen (HLA), which makes them the most promising target for tumor vaccines.^252^, ^253^, ^254^, ^255^, ^256^, ^257^, ^258^, ^259^ Compared with a TAA‐targeted vaccine, a neoantigen‐based vaccine provides inspiring advantages: first, due to the constrained expression of neoantigens in tumor cells only, neoantigen vaccines elicit a true tumor‐specific T‐cell response, and avoid off‐target damage to nontumorous tissues. Next, a substantial fraction of nonsynonymous neoantigens causes strong immunogenicity,^260^ this is perhaps due to the fact that somatic mutated neo‐epitopes manage to bypass the central tolerance. Lastly, prolonged T cell responses, along with immune memory, can be induced by neoantigens. They together prevent potential tumor recurrence and metastasis.^261^, ^262^, ^263^, ^264^

Each tumor patient has their own unique neoantigen profile, also known as the mutanome. With the wide application of next‐generation sequencing and the development of neoantigen prediction technology, each patient can benefit from individualized sequencing for quick and efficient neoantigen identification and selection.^265^, ^266^ Then, a monoepitope or, more commonly, polyepitope mRNA vaccine targeting neoantigens can be rapidly prepared. At present, the clinical results for several neoantigen‐targeting mRNA vaccines are encouraging.^23^, ^25^, ^267^, ^268^

In 2017, there were two significant medical breakthroughs of individualized vaccines for melanoma treatment. Both studies were based on whole‐exome sequencing of tumor tissues. In a study from Harvard Medical School, six melanoma patients received a neoantigen vaccine adjuvanted with an immunostimulant, poly‐ICLC. The results suggested that four patients showed no recurrence within a median of 25 months (from 20–32 months), and two other patients with lung metastases achieved complete remission after relapse by taking the immune checkpoint inhibitor Keytruda (NCT01970358).^253^ At BioNTech, researchers prepared individualized mRNA neoantigen vaccines for each patient. After treatment, the recurrence and metastasis of melanoma were significantly reduced, and the progression‐free survival (PFS) of patients was significantly extended.^25^

Inspired by these results, 21 clinical trials of mRNA tumor vaccines targeting neoantigens are currently under development, according to ClinicalTrials.gov (Table 3). Bioenterprises such as Moderna and BioNTech are actively exploiting customized tumor vaccine in competition with each other. The mRNA‐4157 vaccine developed by Moderna can encode up to 34 neoantigens and has completed a phase I clinical trial (NCT03313778) for the treatment of solid tumors. A combination therapy of the mRNA‐4157 vaccine and the immune checkpoint inhibitor pembrolizumab revealed antitumor potential against a variety of solid tumors.^269^ The phase II clinical trial of mRNA‐4157 vaccine for melanoma has begun to recruit patients (NCT03897881).^229^ Another completed phase I clinical trial (NCT03289962) is BioNTech's combination therapy for the treatment of locally advanced or metastatic solid tumors (e.g., NSCLC, colorectal cancer, melanoma, and triple negative breast cancer). This therapy combines personalized mRNA cancer vaccine BNT122 with the PD‐L1 inhibitor atezolizumab. Among 108 patients who received at least one postdose assessment, the objective response rate (ORR) was about 8% (nine patients), and the SD rate was 49% (53 patients).^270^ NEO‐PV‐01, another personalized neoantigen vaccine for advanced solid tumors, is also being tested in combination with nivolumab. The phase Ib clinical trial (NCT02897765) suggested that, for vaccinated patients with melanoma, NSCLC, and bladder cancer, the corresponding ORRs were 59%, 39%, and 27%; the median PFS was 23.5 months, 8.5 months, and 5.8 months, respectively. Furthermore, the 1‐year OS rates were 96% for melanoma, 83% for NSCLC, and 67% for bladder cancer, all of which were superior to the historical data obtained from programmed death‐1 inhibitor monotherapy.^271^ The existing clinical data suggest that in the treatment of tumor types with a high mutational burden (e.g., melanoma and NSCLC) and mismatch repair‐deficient colorectal cancer, neoantigen vaccines used in combination with immune checkpoint inhibitors should be the focus of future research, because they may provide synergistic effects to enable a higher response rate and PFS.^228^, ^258^, ^259^, ^271^

Although neoantigen vaccines have great prospects, the design of such vaccines depends heavily on the continuous optimization of algorithms, including HLA typing, binding strength of neoantigens and MHC, and T cell receptor (TCR) analysis. They are further limited by the median time from screening neoantigens to vaccine preparation of 103 days (ranging from 89 to 160 days), which is too long for patients with advanced tumors who urgently demand individualized treatment.^25^, ^251^, ^272^, ^273^, ^274^ Despite these difficulties, mRNA vaccines targeting tumor neoantigens may enable in a new era of personalized treatment.

### 5.3 mRNA‐transfected DC vaccines

DC vaccines represent another type of popular cancer vaccine. DCs are ex vivo transfected with TAA‐encoding mRNA in various ways and are then adoptively transferred back. The world's first human clinical trial of mRNA‐transfected DC vaccines was carried out in 2001.^275^ After two decades, there are now 23 clinical trials of DC tumor vaccines prepared by mRNA transfection (Table 4), many of which experience simultaneous transfection with mRNAs encoding multiple TAAs.^264^ In 2010, the US Food and Drug Administration approved the world's first DC tumor vaccine, Sipuleucel‐T, for the treatment of advanced prostate cancer, starting a new chapter for antitumor vaccines.^276^, ^277^, ^278^, ^279^ Efficient loading of tumor antigens into DCs has been one of the core issues in the preparation of DC vaccines. Progress in the in vitro synthesis of mRNA considerably enhances the stability of mRNA, while reducing its immunogenicity by mRNA modification. Therefore, transfecting DCs with TAA‐encoded mRNA is a suitable way to load tumor antigens and has shown great application prospects.^280^, ^281^, ^282^

**TABLE 4:** Currently clinical trials with mRNA transfected dendritic cells tumor vaccines

NCT number	Cancer type	Phases	mRNA encoding for	Interventions	Status	Route	Completion
NCT00639639	Malignant neoplasms of brain	I/II	cmvpp65	Tetanus toxoid; therapeutic autologous dendritic cells; herapeutic autologous lymphocytes	Active, not recruiting	i.d.	2019/12
NCT03615404	Glioblastoma; malignant glioma; medulloblastoma recurrent; pediatric glioblastoma multiforme; pediatric brain tumor	I	pp65	CMV‐DCs with GM‐CSF; Td (tetanus toxoid)	Completed	Null	2020/7/2
NCT02709616	Glioblastoma	I	Multiple TAAs	Personalized cellular vaccine	Completed	i.d./i.v.	2020/6
NCT02808416	Brain Cancer; Neoplasm Metastases	I	Multiple TAAs	Personalized cellular vaccine	Completed	Null	2020/9/1
NCT03334305	Malignant glioma; high grade glioma	I	Autologous tumor‐mRNA	TTRNA‐DC vaccines with GM‐CSF; Dose‐intensified TMZ; autologous hematopoietic stem cells (HSCs); TTRNA‐xALT; Td vaccine	Recruiting	i.d.	2026/5
NCT03396575	Diffuse intrinsic pontine glioma (DIPG); brain stem glioma	I	Autologous tumor‐mRNA	TTRNA‐DC vaccines with GM‐CSF; TTRNA‐xALT; cyclophosphamide + fludarabine lymphodepletive conditioning; dose‐intensified TMZ; Td vaccine; autologous hematopoietic stem cells (HSC)	Recruiting	i.d.	2024/6
NCT01456104	Melanoma	I	tyrosinase‐related peptide 2 (TRP2)	Langerhans‐type dendritic cells (a.k.a. Langerhans cells or LCs)	Active, not recruiting	i.d.	2019/10/1
NCT01995708	Multiple myeloma	I	CT7; MAGE‐A3; WT1	CT7, MAGE‐A3, and WT1 mRNA‐electroporated langerhans cells (LCs)/Standard of care	Active, not recruiting	i.d.	2019/11/1
NCT03927222	Glioblastoma	II	cmvpp65	Human CMV pp65‐LAMP mRNA‐pulsed autologous DCs containing GM‐CSF; temozolomide; tetanus‐diphtheria toxoid (Td); GM‐CSF; 111‐Indium‐labeling of cells for in vivo trafficking studies	Suspended	i.d.	2023/12/1
NCT03688178	Glioblastoma	II	cmvpp65	Human CMV pp65‐LAMP mRNA‐pulsed autologous DCs; temozolomide; varlilumab; Td; 111In‐labeled DCs; unpulsed DCs; HIV‐Gag mRNA‐pulsed autologous DCs	Recruiting	i.d.	2025/3/1
NCT02465268	Glioblastoma multiforme; malignant glioma; astrocytoma, grade IV	II	pp65	pp65‐shLAMP DC with GM‐CSF; unpulsed PBMC and saline; Td; saline; pp65‐flLAMP DC with GM‐CSF	Recruiting	i.d.	2024/6
NCT02692976	Prostatic Neoplasms	II	NY‐ESO‐1; MUC1; PepTivator	mDC vaccination; pDC vaccination; mDC and pDC vaccination	Completed	i.t.	2019/3/6
NCT03083054	Myelodysplastic syndromes; AML	I/II	WT1	Autologous dendritic cells electroporated with WT1 mRNA	Active, not recruiting	Null	2020/7/1
NCT02649829	Malignant pleural; mesothelioma	I/II	WT1	Dendritic cell vaccination plus chemotherapy	Recruiting	i.d.	2021/11
NCT04911621	Glioma; diffuse intrinsic pontine glioma	I/II	WT1	Dendritic cell vaccination + temozolomide‐based chemoradiation; Dendritic cell vaccination ± conventional next‐line treatment	Recruiting	i.d.	2027/6
NCT02649582	Glioblastoma multiforme of brain	I/II	WT1	Dendritic cell vaccine plus temozolomide chemotherapy	Recruiting	i.d.	2022/12
NCT01885702	Colorectal cancer	I/II	CEA	DC vaccination	Active, not recruiting	Null	2019/6
NCT04567069	Gastric cancer	I/II	MG‐7	MG‐7‐DC vaccine; CTL; Sintilimab Injection	Recruiting	s.c.	2022/6
NCT02528682	Hematological malignancies	I/II	minor histocompatibility antigens (MiHA)	MiHA‐loaded PD‐L‐silenced DC vaccination	Completed	i.v.	2019/7/1
NCT01334047	Recurrent epithelial; ovarian cancer	I/II	survivin;hTERT; cancer stem cell mRNA	DC‐006 vaccine	Terminated	Null	2022/4
NCT01197625	Prostate cancer	I/II	survivin; hTERT; cancer stem cell mRNA	Dendritic cell vaccine	Active, not recruiting	Null	2025/9
NCT03548571	Glioblastoma	II/III	survivin;hTERT; cancer stem cell mRNA	Dendritic cell immunization; adjuvant temozolomide	Recruiting	i.d.	2023/5
NCT05000801	Acute myeloid leukemia	NA	WT1; hTERT; survivin	DC vaccine (dendritic cells loaded with tri‐antigens (WT1/hTERT/survivin)	Recruiting	Null	2026/7

*Note*: The table summarizes the currently clinical trials with mRNA transfected dendritic cells tumor vaccines registered at *Clinical Trials.gov*.

Abbreviations: AML, acute myeloid leukemia; CEA, carcinoembryonic antigen; CMV,cytomegalovirus; CTL, cytotoxic T lymphocyte; DC, dendritic cell; GM‐CSF, granulocyte‐macrophage colony stimulating factor; i.d., intradermal injection; i.t., intradermal injection; i.v., intravenous injection; MG‐7, Ag monoclonal gastric cancer 7 antigen; mRNA, messenger ribonucleic acid; NA, not available; s.c.,subcutaneous injection; TTRNA‐DC: total tumor RNA‐dendritic cell.

The function of DCs is collectively determined by the balance of several factors, such as cell surface co‐stimulatory molecules, co‐inhibitory molecules, and activating and inhibitory cytokines. Therefore, DC vaccines designed to boost multiple aspects of DC function are likely to achieve optimal efficacy. The immunostimulatory activity of DC is elevated remarkably when it is electroplated with mRNAs that encode adjuvants, such as OX40L or 4‐1BBL.^283^, ^284^ Another representative approach is the TriMix platform developed by eTheRNA. TriMix includes three mRNA components that encode the adjuvants CD70 and CD40L, and constitutively active TLR4.^101^, ^105^, ^285^ Co‐transfection of TriMix and mRNA encoding the HLA‐II targeting signal (DC‐LAMP) and the melanoma‐associated antigen fusion protein (MAGE‐A3, MAGE‐C2, tyrosinase or gp100) into autologous DC to prepare a DC vaccine (TriMixDC‐MEL) activation and promote DC maturation and function is underway (NCT01066390).^286^ In a further clinical trial of TriMixDC‐MEL in combination with ipilimumab (TriMixDC‐MEL IPI), a 6‐month disease control rate of 51% was observed. After 5 years of clinical follow‐up, vaccine‐induced immune responses were evaluated in 15 patients (NCT01302496) and were sustained in 12 of the 15 patients. Stronger and more extensive immune responses were detected in patients with complete and partial responses compared to patients with stable and progressive disease.^105^, ^287^

The advantages of DC vaccines include controllable ex vivo culture conditions of DC cells, accurate tumor antigen loading by mRNA transfection, and high transfection efficiency. However, it also shares some problems common to all cell vaccines that require individualization, including a long production cycle, high labor costs, and poor tumor targeting. These issues need to be addressed before general application is feasible.

## 6 CONCLUSIONS AND PERSPECTIVES

mRNA vaccines have been developed at an unprecedented speed. As outlined in this review, although immuno‐oncologists were among the first to show early and wide acceptance of mRNA‐based vaccines, they can also be a generalized prophylactic measure for infectious diseases and have become the most promising approach to COVID‐19 prevention and control.

There are two key aspects that allow the rapid pace of mRNA vaccine development: the mRNA sequence design and in vivo delivery systems.^110^, ^288^, ^289^, ^290^, ^291^ Because the therapeutic use of mRNA vaccine seems more feasible, owing to technical innovations that greatly improved the mRNA delivery efficiency and mRNA stability in the physiological environment, this potential billion‐dollar market has attracted funding from diverse sources, including governments, research project agencies, and pharmaceutical enterprises. Moderna Therapeutics, the “unicorn” set up in 2010, has already raised more than US $2 billion for the development and commercialization of mRNA‐based vaccines.^292^

There are several aspects to mRNA vaccines that require further improvements. First, adverse effects should be harnessed, partly by introducing novel lipids that are more biocompatible, for example, ready to be fully catabolized into harmless substances in the body.^293^, ^294^, ^295^ Current ionizable cationic lipid and PEGylated lipid components can be substituted.^26^ Second, targeted delivery of mRNA vaccine into DCs should be put to real‐world use to enhance efficacy, reduce dosage, and limit adverse effects. Vehicles, such as LNPs, can be endowed with targeting ability by introducing functionalized components.^20^, ^295^ Novel intravital administration techniques can be developed. Third, novel adjuvants should be incorporated into the formulation to further boost the efficacy of the mRNA vaccines.^29^, ^296^, ^297^ A delicate balance of several factors, for example, stimulation of immunity, exacerbated inflammation, and suppression of mRNA expression is required. Fourth, the issue raised by nucleoside modification and codon optimization of mRNA needs to be addressed to ensure efficient and accurate translation of full‐length polypeptides that are properly folded.^49^, ^298^, ^299^, ^300^ Fifth, mRNA cancer vaccines in combination with other immunotherapies should be investigated to determine which combinations exert synergetic effects in cancer treatment.^66^, ^241^, ^301^, ^302^ Lastly, it is important to determine whether mRNA vaccines are processed and function in the same way in animal models and in humans, because some mRNA vaccines, which showed immunogenicity in preclinical animal trials, have generated disappointing results in human trials.^28^, ^91^, ^175^, ^303^

## CONFLICT OF INTEREST

The authors declare there is no conflict of interest.

## AUTHOR CONTRIBUTIONS

X.Z. conceived the ideas of the manuscript. Y.Z.G., J.Y.D., N.Y. and Y.X.Y. searched and screened the literature, wrote the draft, and drew the figures and tables. Y.Z.G. and X.Z. critically revised the article, tables, and figures, supervised the coordination, and X.Z. was in charge of correspondence. All authors approved the final submitted version of this manuscript.

## ETHICS STATEMENT

No ethical approval was required for this study.

## Data Availability

Data sharing is not applicable to this article as no new data were created or analyzed in this study.
